# Tibial bone defect prediction based on preoperative artefact-reduced CT imaging is superior to standard radiograph assessment

**DOI:** 10.1007/s00167-023-07527-4

**Published:** 2023-08-10

**Authors:** Marco Brenneis, Dimitrios A. Flevas, Troy D. Bornes, Sebastian Braun, Andrea Meurer, Peter K. Sculco, Fernando J. Quevedo-González, Friedrich Boettner

**Affiliations:** 1 Stavros Niarchos Foundation Complex Joint Reconstruction Center, Hospital for Special Surgery, New York, NY USA; 2 Adult Reconstruction and Joint Replacement Service, Hospital for Special Surgery, New York, NY USA; 3 Department of Biomechanics, Hospital for Special Surgery, New York, NY USA; 4 Department of Orthopaedics (Friedrichsheim), University Hospital, Goethe University Frankfurt, Frankfurt/Main, Germany; 5 Division of Orthopaedic Surgery, Royal Alexandra Hospital, University of Alberta, Edmonton, Canada; 6 Medical Park Klinik, Bad Wiessee, Germany

**Keywords:** 3D-CT, Defect classification, AORI, Zonal fixation, Volumetric defect measurement, Revision total knee arthroplasty

## Abstract

**Purpose:**

The purpose of this study was to evaluate the accuracy of preoperative CT-based Anderson Orthopaedic Research Institute (AORI)-grading and to correlate Computed tomography (CT)-based volumetric defect measurements with intraoperative AORI findings.

**Methods:**

99 patients undergoing revision total knee arthroplasty (rTKA) with preoperative CT-images were identified in an institutional revision registry. CT-image segmentation with 3D-Slicer Software was used to create 3D tibial bone defects which were then graded according to the AORI-classification. The AORI classification categorizes tibial defects into three types: Type I has healthy cortical and cancellous bone near the joint line, Type II involves metaphyseal bone loss affecting one or both condyles, and Type III indicates deficient metaphyseal bone with distal defects and potential damage to the patellar tendon and collateral ligament attachments. These 3D-CT gradings were compared to preoperative X-ray and intraoperative AORI grading. The Friedman test was used to investigate differences between AORI values of each measurement method. Volumetric 3D-bone defect measurements were used to investigate the relationship between AORI classification and volumetric defect size in the three anatomic zones of the tibia.

**Results:**

Substantial agreements between preoperative 3D-CT AORI and intraoperative AORI (kappa = 0.663; P < 0.01) and fair agreements between preoperative X-ray AORI and intraoperative AORI grading (kappa = 0.304; P < 0.01) were found. Moderate correlations between volume of remaining bone and intraoperative AORI grading were found in epiphysis (r_S_ = – 0.529; P < 0.001), metaphysis (r_S_ = – 0.557; P < 0.001) and diaphysis (r_S_ = – 0.421; P < 0.001). Small volumetric differences between AORI I vs. AORI II defects and relatively large differences between AORI II and AORI III defects in each zone were detected.

**Conclusion:**

Tibial bone defect prediction based on preoperative 3D-CT segmentation showed a substantial agreement with intraoperative findings and is superior to standard radiograph assessment. The relatively small difference in defect volume between AORI I, IIa and IIb suggests that updated CT-based classifications might hold benefits for the planning of rTKA.

**Level of evidence:**

Retrospective Cohort Study; III

## Introduction

Revision total knee arthroplasty (rTKA) is faced with particular challenges due to the presence of bone defects and limited bone stock [25]. In order to achieve durable fixation, the size and location of tibial bony defects influences the use of cones/sleeves, augments, and length of the stem [28]. As such, being able to quantify bone defects preoperatively, allows for more effective planning prior to rTKA. [10, 26]. The concept of zonal fixation, published by Morgan-Jones et al., provides a methodology to plan rTKA and achieve durable fixation [19]. According to this system, a primary and long-term implant stability can be obtained when fixation in at least two of the epiphyseal, metaphyseal, and diaphyseal zones is achieved. However, developing a treatment algorithm for complex revision cases requires a reliable classification system for assessing bone loss preoperatively [12, 27].

The Anderson Orthopaedic Research Institute (AORI) classification is one of the most commonly used algorithms to describe bone loss in rTKA [7, 12]. The AORI classification was published in 1999 with the intention to classify tibial and femoral bone loss pre- and intraoperatively to guide surgical treatment [7]. In a recent study, Bole et al. categorized tibial defects in rTKA according to their shape using plain radiographs [3]. However, such two-dimensional analysis was performed on post-revision radiographs and provided only limited information about the three-dimensional (3D) volume of the defects. On the other hand, advancements in computed tomography (CT) technology, including improvements in multi-detector CTs and modern metal artifact reduction protocols has significantly reduced beam-hardening artifacts [13, 24]. As a consequence, analysis of CT images is now possible, even in the presence of metal components, allowing 3D analysis of bone defects and volumetric measurements from cross-sectional imaging obtained pre-rTKA [4, 5, 29, 31].

Therefore, the goal of the current study was to evaluate the preoperative CT-based 3D analysis of bone defects in rTKA. The following research questions were addressed: (1) Are preoperative CT-scans superior to plain radiographs in predicting the AORI grade of defects encountered at the time of rTKA? (2) Does the intraoperative AORI grading correlate with preoperative CT-based volumetric defect measurements in the three anatomic zones of the tibia? It was hypothesized that the intraoperative AORI defect grading will show greater correlation with preoperative CT based grading compared to plain radiographs.

## Materials and methods

This single center, retrospective study was approved by the institutional review board. All investigations were performed in accordance with relevant guidelines and regulations.

Using a prospectively maintained institutional database, 144 patients (144 knees) who underwent revision TKA between 2017 and 2022 were identified. Patients were included if they had preoperative CT imaging, AP and lateral radiographs, and if bone defects were graded intraoperatively according to the AORI classification. Patients were excluded if the tibial component was not removed (N = 1) or if the imaging was not suitable for evaluation (n = 44) due to excessive motion artifacts or incomplete visualization of the region of interest because the scan length was not sufficient to evaluate the entire periprosthetic bone region, including the epiphyseal, metaphyseal, and diaphyseal zones. In case of excessive motion artifacts (blurring, streaking, or distortions that appear in the reconstructed CT images due to patient motion during the scanning process) the CT images were evaluated by the observers and excluded by consensus. Ultimately, 99 patients (99 knees) were included within the study. Primary reasons for revision included infection (N = 20, 20.2%), aseptic loosening (N = 33, 33.3%), instability (N = 10, 10.1%), osteolytic lesions (N = 10, 10.1%), periprosthetic fracture (N = 8, 8.1%), or stiffness/arthrofibrosis (N = 18, 18.2%).

All CT-scans were obtained with a Discovery CT750 HD scanner (GE Healthcare), using similar scanning parameters (0.625 mm slice-thickness; 140 kVp, and 300–310 mA; mean CTDIvol 55.54 ± 7.75 mGy). In case of bilateral implanted knee prosthesis, the contralateral leg was offset (flexed) to reduce the effect of metal artifacts from the opposite side. A 6th year orthopedic resident (MB) under the supervision of a board-certified orthopedic surgeon (FB) segmented the tibia and fibula, tibial implant, cement, and bony defect from the preoperative CT-scans using a metal artifact reduction reconstruction (3D Slicer v5.1.0, Boston, MA) [8]. During the implementation of the 3D reconstruction algorithm, 3D reconstructions were performed on 5 randomly selected CT images and standard bone reconstructions were compared with metal artifact reduction reconstructions (MAR (GE)—no dual energy). This pilot test revealed that 3D reconstructions and manual refinements were best performed on metal artifact reduced sequences. The projection-based artifact correction (MAR) is based on a combination of corrected iterative data and raw data and has been shown to improve the visibility of tissues adjacent to and distant from the implant and is superior in comparison to conventional reconstruction [6, 20]. A disadvantage of metal artifact-reduced sequences is that new artifacts induced by these projection-based algorithms commonly appear at the bone-metal interface [16]. Those artifacts can be incorrectly interpreted as osteolysis. In order to prevent an overestimation of the defects the projection-based corrected images were compared side-by-side with the standard CT images in borderline cases and manual refinements were adjusted accordingly.

**Fig. 1:**
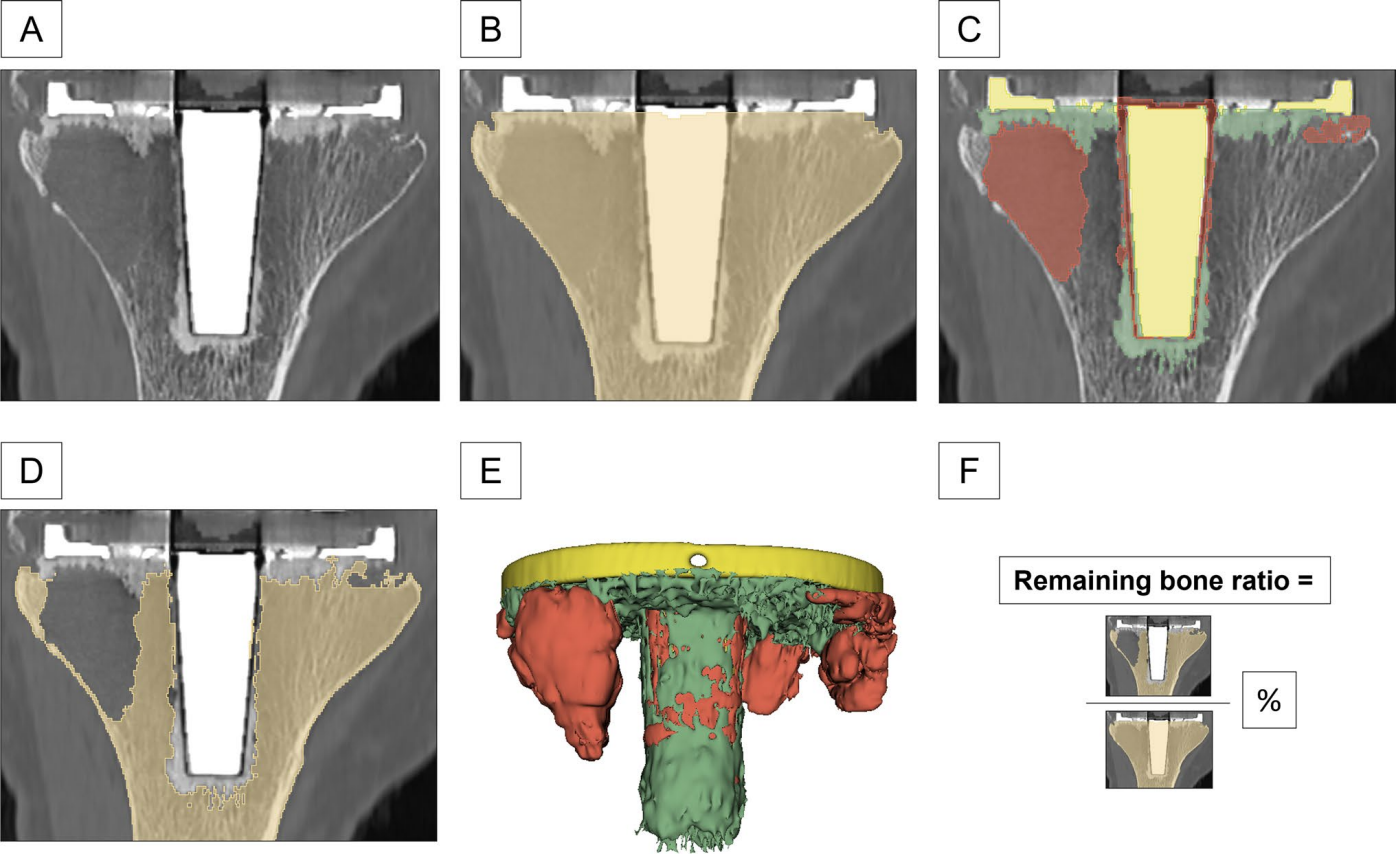
Three-dimensional defect segmentation.** A** Preoperative 3D-CT scan of a big epi- and metaphyseal defect in anterior–posterior view. **B** The total tibial bone (CT volume between the most distal part of the depicted tibia and the tibial cut) was defined as region of interest (ROI). **C** The volume of that ROI was separated in bone (light brown), implant (yellow), cement (green) and defect (red). **E** The total defect volume (cm^3^) was calculated by adding the volumes of the segmented implant, the cement, and the defect. **D** The volume of the remaining tibial bone after removal of the implant was obtained by subtracting the total defect from the tibial bone. Each segmentation was performed in three dimensions. Remaining bone ratio was calculated by dividing the ratio of the remaining tibial bone volume and the total tibial bone volume (**F**)

An initial segmentation was obtained by considering specific thresholds for the Hounsfield Units (HU) of the tibial defect (range – 1024 to 200), the tibia and fibula (range 200–1800), the tibial cement (range 300–1400), and the tibial implant (range 2000–3071) [1]. These initial segmentations were manually refined to obtain separate, non-overlapping and complementary volumes (cm^3^) for the tibial defect, bone, cement, and implant [31] (Fig. 1A–C). Subsequently, the total tibial bone (CT volume between the tibial cut and the most distal part of the depicted tibia) as region of interest (ROI) was identified (Fig. 1B). The volume of that ROI was separated in bone, cement, implant (note that this is not the total implant volume, but just the implant volume within the ROI), and defect (Fig. 1C). The volume of the total defect (in cm^3^) was then calculated as implant + cement + defect (Fig. 1E). Finally, the volume of the remaining tibial bone after removal of the prosthesis was obtained by subtracting the total defect from the total tibial bone (Fig. 1D). To allow comparison across bones of different sizes, the ratio of the remaining tibial bone volume (the bone volume minus the defect) was computed (ratio of the remaining tibial bone volume divided by the total tibial bone volume) (Fig. 1E).

**Fig. 2:**
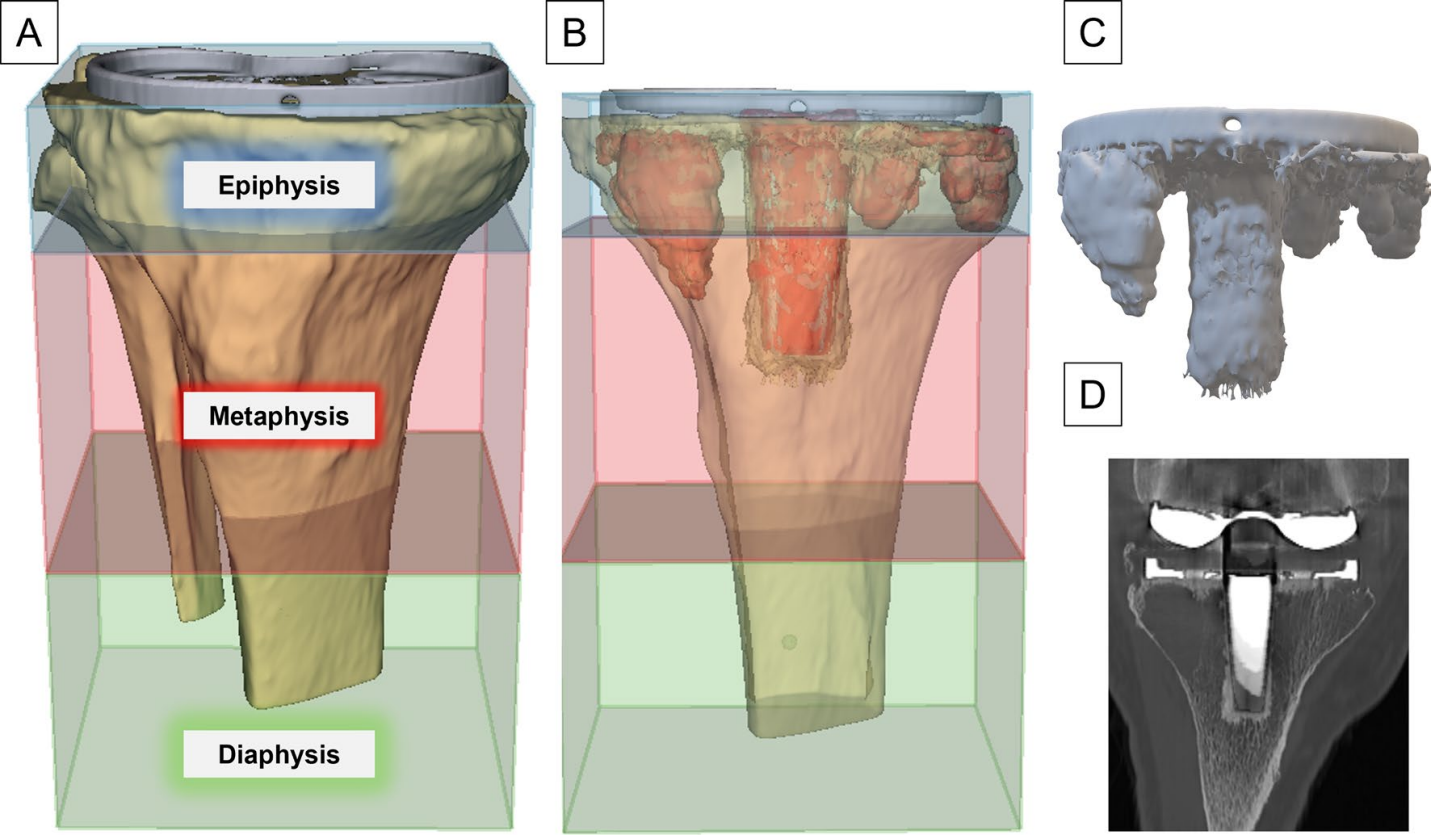
Three-dimensional segmentation of tibial bone defects. **A** Tibial epiphysis (blue) and metaphysis (red) are defined by a square with a side-length that is the same as the widest part of the tibial epiphysis. Bone outside of this square is considered to be diaphysis (green). **B** To determine the volume of the remaining tibial bone after removal of the prosthesis, the defect was subtracted from the tibial bone. **C** Segmented 3D model of the defect. **D** Coronal plane of the respective CT

In addition, the ROI was subdivided according to the concept of zonal fixation into epiphyseal, metaphyseal, and diaphyseal zones following the rule of the square [19, 22], where the metaphyseal-diaphyseal junction is defined by a square with a side-length equal to the widest part of the epiphysis. The epiphyseal-metaphyseal junction was defined at the widest mediolateral point of the fibular head (Fig. 2) [2]. The volume of the remaining bone in each of these areas was computed relative to the total tibial volume of the zone.

The 3D defects and the preoperative X-rays (anterior–posterior view and lateral view) were graded according to the well-established AORI classification by two graders (MB and SB [orthopaedic surgeon]). It is a categorical bone loss assessment instrument published by Engh et al. in 1999 [7]. In total, three categories are described: A tibial AORI type I defect has healthy and sustainable cortical as well as cancellous bone near the original joint line. A tibial AORI type II defect is described as a cancellous metaphyseal bone loss involving one condyle (AORI IIa) or both condyles (AORI IIb). In contrast to AORI type I defects, AORI type II defects cross a fictitious plane through the tip of the fibular head. Tibial AORI Type III defects describe deficient metaphyseal bone with bone defect or component subsidence distal to the tibial tubercle and possible damage to the patellar tendon and collateral ligament attachments.

There was a high interobserver agreement when grading the defect based on preoperative Xray- (kappa = 0.892; p < 0.001) and 3D-CT AORI grading (kappa = 0.918; p < 0.001). Any disagreement in the grading was resolved by consensus. The graders were blinded to the intraoperative AORI grading. The agreement between the two observers as well as the agreement between the gradings of the 3D defects and the prospectively collected intraoperative AORI gradings was assessed with a categorical analysis using the Cohen’s kappa. The criteria of Landis and Koch were used in the assessment of the results (range 0.01–0.20 slight; range 0.21–0.40 fair; range 0.41–0.60 moderate; range 0.61–0.80 substantial and range 0.81–1.00 almost perfect agreement) [15]. Furthermore, AORI gradings were assigned to an ordinal scale (AORI I = 1; AORI IIa = 2; AORI IIb = 3; AORI III = 4) to analyze if CT and X-ray based assessments categorized defects into similar severity compared to intraoperative assessments.

The study also evaluated the relationship between AORI classification and defect size, following the concept of zonal fixation. The ratios of remaining bone in the epiphyseal, metaphyseal and diaphyseal zones was analyzed in relation to the AORI classification using the Spearman’s rank correlation analysis (r_s_). A sensitivity power analysis was calculated in order to evaluate what effect sizes a within-subjects design is sensitive enough to detect. A Spearman's rank correlation coefficient with 99 participants would be sensitive to effects of r_S_ = 0.28 with 80% power (alpha = 0.05, two-tailed). The ratios of remaining bone across all AORI classifications were compared using the Kruskal–Wallis test. The Friedman test was used to investigate differences between AORI values of each measurement method.

Statistical analysis was performed using SPSS version 26 (IBM Corporation, New York). The Shapiro–Wilk test was used to test normal distribution of the analyzed parameters. The significance level was set at p < 0.05. All tests are two-sided.

## Results

### Correlation between preoperative CT and plain radiograph grading and intraoperative AORI classification

**Fig. 3:**
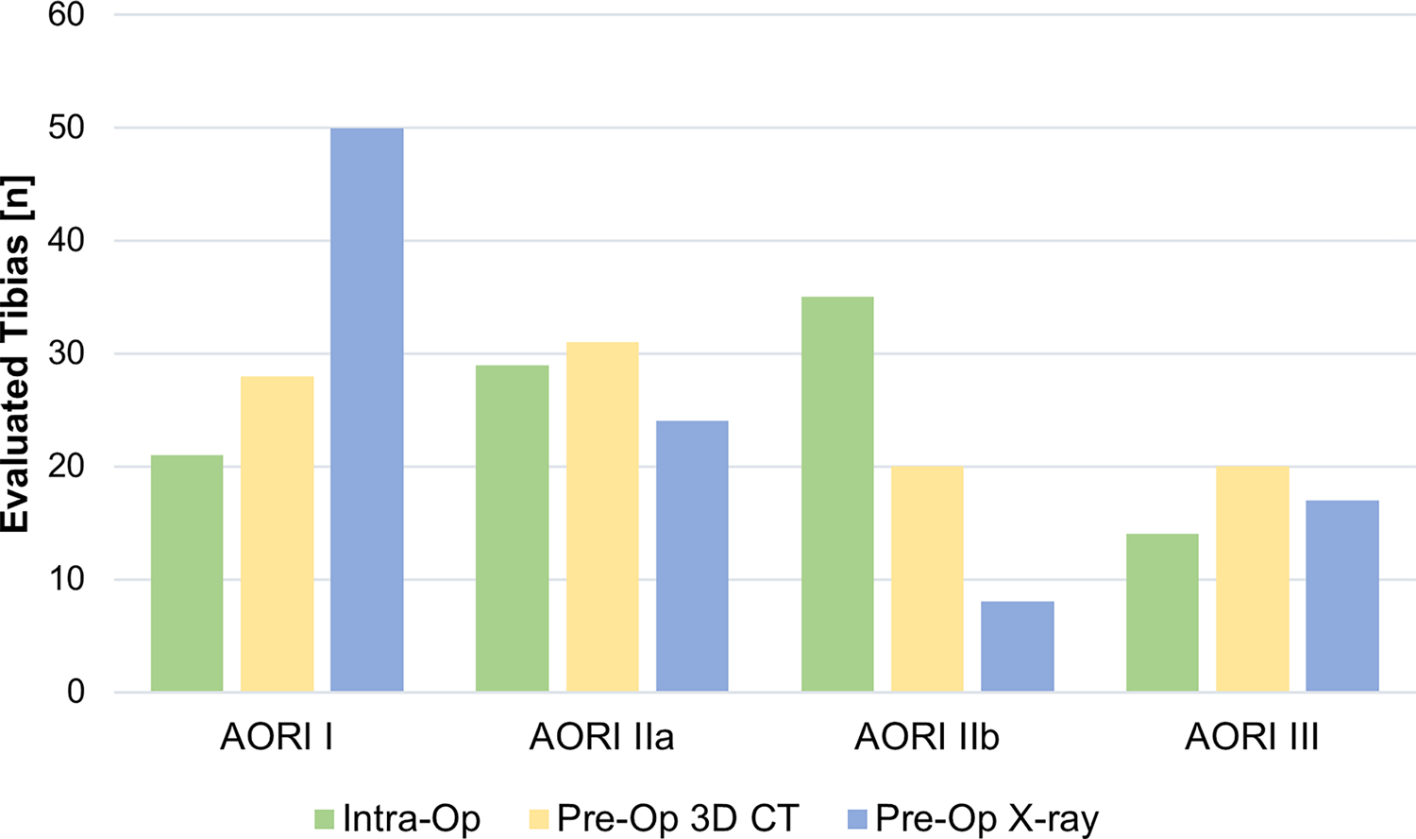
Distribution of the preoperative X-ray, the preoperative 3D CT and intraoperative AORI grading. Histogram of the respective AORI gradings. Data represent the number of investigated knees [n]

Intraoperatively, 21 (21.2%) defects were graded as AORI I, 29 (29.3%) as AORI IIa, 35 (35.4%) as AORI IIb and 14 (14.1%) as AORI III (Table 1). Classifications based on preoperative 3D-CT segmentation and preoperative X-rays are shown in Fig. 3. The correlation with intraoperative AORI findings was stronger for the preoperative 3D-CT AORI grading (r_s_ = 0.75, p < 0.001) than for the preoperative X-ray AORI grading (r_s_ = 0.56, p < 0.001). The preoperative 3D-CT AORI assessment showed a substantial agreement (kappa = 0.66; p < 0.001) with intraoperative AORI assessment. In contrast, the agreement between preoperative X-ray AORI assessment and intraoperative AORI assessment was fair (kappa = 0.304; p < 0.001). Preoperative CT correctly predicted 17 (48.6%) IIb defects and X-ray correctly predicted 5 (14.3%) IIb defects out of a total of 35 intraoperative IIb defects. The results of the comparison of the respective methods were shown in Table 1.

**Table 1:** Level of agreement between intraoperative, preoperative 3D-CT and peroperative X-ray AORI grading

IntraOP	PreOP 3D-CT					PreOP X-ray				
	AORI I	AORI IIa	AORI IIb	AORI III	Total	AORI I	AORI IIa	AORI IIb	AORI III	Total
AORI I	21(100%)	0(0%)	0(0%)	0(0%)	21	20(95.2%)	1(4.8%)	0(0%)	0(0%)	21
AORI IIa	1(3.4%)	23(79.3%)	3(10.3%)	2(6.9%)	29	14(48.3%)	10(34.5%)	3(10.3%)	2(6.9%)	29
AORI IIb	6(17.1%)	7(20%)	17(48.6%)	5(14.3%)	35	15(42.9%)	11(31.4%)	5(14.3%)	4(11.4%)	35
AORI III	0(0%)	1(7.1%)	0(0%)	13(92.9%)	14	1(7.1%)	2(14.3%)	0(0%)	11(78.6%)	14
Total	28(28.3%)	31(31.3%)	20(20.2%)	20(20.2%)	99	50(50.5%)	24(24.2%)	8(8.1%)	17(17.2%)	99

**Fig. 4:**
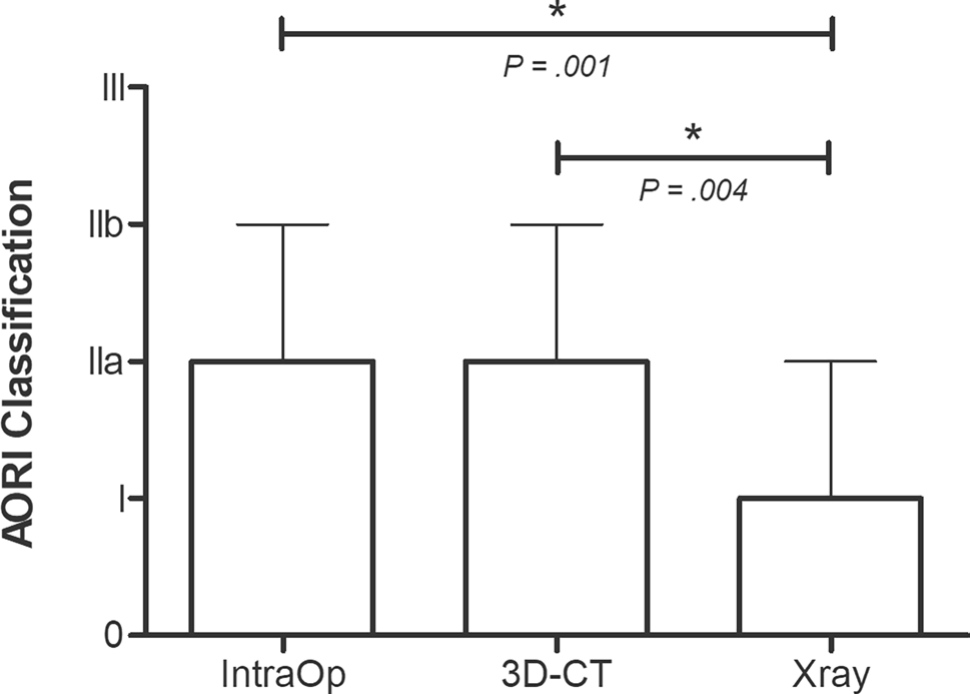
Comparison of preoperative AORI gradings using a preoperative 3D-CT model and X-rays. Preoperative AORI grade was assessed using the created preoperative 3D-CT defect model and the preoperative X-rays. Intraoperative AORI grade was assessed at the time of surgery. Data represent medians with interquartile range

The preoperative X-ray assessment graded defects less severely (median = 1, interquartile range (IQR) = 1–2) (p = 0.001) than intraoperative AORI assessment (median = 2, IQR = 2–3) and preoperative 3D-CT classification (median = 2, IQR = 1–3, p = 0.004). No significant difference between preoperative 3D-CT grading and intraoperative AORI grading was found (p = n.s.) (Fig. 4).

### Correlation between intraoperative AORI classification and zonal volumetric defect measurements

**Fig. 5:**
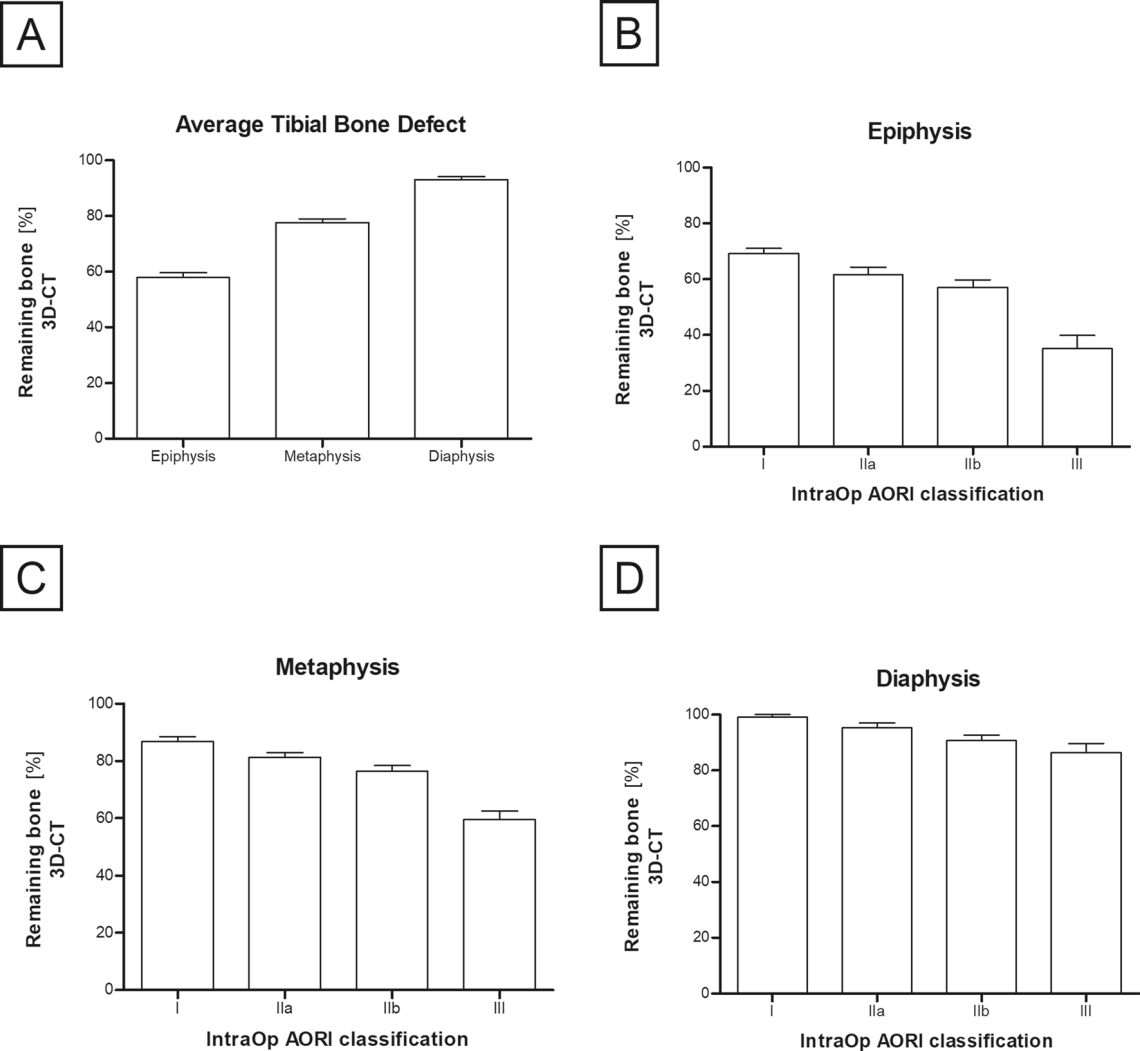
Remaining bone [%] of the respective tibial zones (**A**) with special focus on intraoperative AORI classification (**B**–**D**). Remaining bone [%] is defined as the ratio of “remaining bone” volume to initial tibial bone volume in CT imaging. Mean remaining bone ratios of the epiphyseal, the metaphyseal and the diaphyseal zone (**A**). Defects were divided in four groups according to intraoperative AORI classification. Remaining bone ratios of epiphyseal (**B**), metaphyseal (**C**) and diaphyseal zone (**D**) were assessed. Data represent means with SEM

The relative volume of the total bone defect in the CT-assessment was larger in the epiphyseal zones (42%) than in the metaphyseal (22%) and diaphyseal zones (7%) (Fig. 5A). No significant differences in the 3D-CT remaining bone ratio of the epiphysis, metaphysis and diaphysis were found between AORI I and AORI IIa (epiphysis: P = n.s., metaphysis: P = n.s., diaphysis: P = n.s.). There was also no difference in the remaining bone ratio in any zone between AORI IIa and AORI IIb defects (epiphysis: P = n.s., metaphysis: P = n.s., diaphysis: P = n.s.), respectively (Fig. 5B–D). In addition, there was no significant difference in remaining diaphyseal bone between intraoperative AORI IIb and AORI III defects (P = n.s.). All remaining group comparisons differed significantly (P < 0.05).

**Fig. 6:**
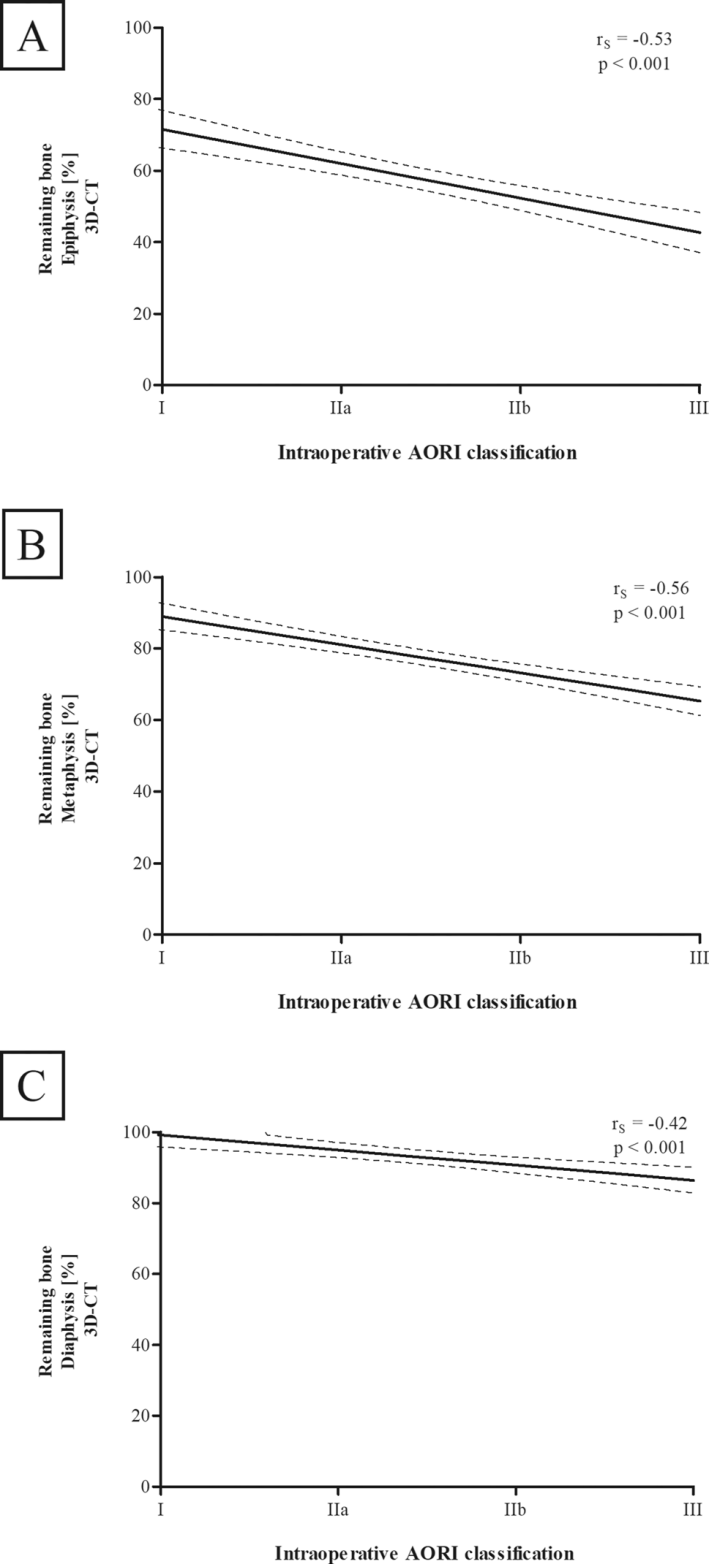
Correlation between 3D-CT defect size and intraoperative AORI classification. Remaining bone [%] of the respective tibial zones (**A** epiphysis, **B** metaphysis, **C** diaphysis) correlates negatively with intraoperative AORI classification. Remaining bone [%] was measured with 3D-CT segmentation and is defined as the ratio of “remaining bone” volume to initial tibial bone volume. Defects were divided in four groups according to intraoperative AORI classification

The intraoperative AORI classification showed a negative correlation (Fig. 6) with the remaining bone ratio at the epiphysis (r_s_ = – 0.53, P < 0.001, N = 99), metaphysis (r_s_ = – 0.56, P < 0.001, N = 99) and diaphysis (r_s_ = – 0.42, P < 0.001, N = 99).

## Discussion

The most important finding of this study was that CT-based tibial bone defect evaluation more accurately predicts the intraoperative findings than the plain radiograph based assessment and that CT-based grading showed substantial agreement with intraoperative AORI results. The differences in defect volume between AORI I, IIa and IIb were relatively small suggesting that CT based grading might be beneficial for implant selection that follows the principles of zonal based reconstruction.

AORI grading on preoperative X-ray’s showed only a fair agreement with intraoperative AORI findings and significantly underestimated the severity of intraoperative bone loss. This is in line with prior studies which also showed low sensitivity of preoperative single-plane and bi-plane X-rays in detecting osteolytic lesions around TKA implants (17% small defects—66% large defects) [14, 21, 24, 29]. Metallic implants often obscure bony lesions and the acquisition of radiographs is highly technician dependent. This is one reason why cross-sectional imaging with modern techniques might be more favorable in predicting bone loss. In this way, the results of the present study revealed that preoperative CT-based grading of tibial bone defects showed greater correlation with intraoperative findings (r_s_ = 0.75, p < 0.001). A recent cadaveric bones study also showed a high sensitivity (83%) and specificity (98%) of CT-based assessments to evaluate tibial bone lesions in the presence of metallic implants [14, 29]. The results of our study show that 3D defect segmentation significantly improves the prediction of intraoperative bone loss according to the AORI classification. Preoperative improved knowledge of the size of tibial bone defects might offer several benefits. It might allow for a more precise surgical planning as well as implant selection and therefore reduce operative time and costs. Furthermore, it might also improve effective patient counseling and surgical outcome. However, some inaccuracies due to implant removal and debridement of non-viable bone persist, especially in the detection of IIb defects.

Additional advantages of 3D imaging are that bone defects, implants, and defect locations can be assessed in more detail, and volumetric defect size measurements can be performed. In the present study the epiphysis, metaphysis and diaphysis was defined according to the "concept of zonal fixation" as well as the “rule of the square” and volumetric measurements in each individual zone were performed [19]. There is a difference noted regarding our definition of zones and the AORI classification. Engh et al. described the epiphysis as a zone above a fictive plane through the tip of the fibular head [7]. In contrast, the current paper used the level of the fibular head with the largest mediolateral width to distinguish between tibial epiphysis and metaphysis [2]. Consequently, the epiphyseal zone in the current paper is larger than originally reported by Engh et al., however, it is also less impacted by bone defects of the tip of the fibular. In order to investigate if the AORI classification adequately addresses the concept of zonal fixation, the ratio of remaining bone was compared with the intraoperative assessed AORI classification and moderate negative correlations in each zone were found. The small differences in defect volume between AORI I vs. AORI II and the relatively large difference between AORI II and AORI III defects suggest that an updated grading for AORI II and III might be beneficial to better facilitate planning according to the concept of zonal fixation. Cones and Sleeves make fixation in the metaphyseal zone more important [23]. These proposed updated grading should take the volumetric size and containment of the defect as well as the bone quality of the respective zones into account [11, 30].

Despite the more accurate prediction of tibial bone defects, CT radiation exposure—especially in young patients—and higher equipment costs must be taken into consideration [18]. In contrast, radiographs are ubiquitous available, inexpensive and can be used to monitor implant position, integrity and stability [17]. Furthermore, 3D segmentation of one CT requires a considerable amount of time for analysis (approximately 45–60 min). This complex and time-consuming segmentation makes integration into the clinical routine difficult. Nonetheless, automation and artificial intelligence could reduce the duration of segmentation and might facilitate its integration into clinical practice [9].

The current study has the following limitations: The CT scans were collected retrospectively. This limited the inclusion of patients to those who got a CT scan preoperatively and may have caused selection bias. The distinction between metal artifacts and defects was occasionally difficult. In borderline cases, possible artifacts were not considered a defect to avoid overestimation of defect sizes. This and the fact that preoperative imaging does not account for additional defects created intraoperative may have led to an underestimation of defect sizes. Furthermore, patients with different implants were investigated. This could have led to limited assessability due to different type of metal artifacts. Nevertheless, a substantial correlation between preoperative defect prediction and intraoperative results could be shown. Continuous optimization of artifact suppression protocols might further increase the accuracy of CT image segmentation. Lastly, only tibial defects were examined. The evaluation of femoral defects might be hampered by higher artifacts due to the shape of the prosthesis. In future studies, accuracy of 3D segmentation must also be investigated for femoral defects.

## Conclusions

In conclusion, tibial bone defect prediction based on preoperative 3D-CT segmentation showed a substantial agreement with intraoperative findings and is superior to standard radiograph assessment. The differences in volumetric defect size between AORI I and II are small. The study suggests that the AORI classification could be updated to include a 3D defect assessment of the individual zones. Future improvements of automation might reduce segmentation time and further improve the visualization and prediction-accuracy of bone defects.

## Data Availability

The data that support the findings of this study are available from the corresponding author, [M.B.], upon reasonable request.

## Abbreviations

3D: Three-dimensional
AORI: Anderson Orthopaedic Research Institute
HU: Hounsfield units
ROI: Region of interest
rTKA: Revision total knee arthroplasty
TKA: Total knee arthroplasty
